# The Anti-Cancer Effect of Linusorb B3 from Flaxseed Oil through the Promotion of Apoptosis, Inhibition of Actin Polymerization, and Suppression of Src Activity in Glioblastoma Cells

**DOI:** 10.3390/molecules25245881

**Published:** 2020-12-12

**Authors:** Nak Yoon Sung, Deok Jeong, Youn Young Shim, Zubair Ahmed Ratan, Young-Jin Jang, Martin J. T. Reaney, Sarah Lee, Byoung-Hee Lee, Jong-Hoon Kim, Young-Su Yi, Jae Youl Cho

**Affiliations:** 1 Department of Integrative Biotechnology, Biomedical Institute for Convergence at SKKU (BICS), Sungkyunkwan University, Suwon 16419, Korea; nakyoon.sung@monash.edu (N.Y.S.); jd279601@gmail.com (D.J.); younyoung.shim@usask.ca (Y.Y.S.); 2 Department of Plant Sciences, University of Saskatchewan, Saskatoon, SK S7N 5A8, Canada; martin.reaney@usask.ca; 3 Guangdong Saskatchewan Oilseed Joint Laboratory, Department of Food Science and Engineering, Jian University, Guangzhou 510632, China; 4 Department of Biomedical Engineering, Khulna University of Engineering and Technology, Khulna 9203, Bangladesh; zubairahmed@bme.kuet.ac.bd; 5 College of Veterinary Medicine, Chonbuk National University, Iksan 54596, Korea; jyj3010@daum.net; 6 National Institute of Biological Resources, Environmental Research Complex, Incheon 22689, Korea; lsr57@korea.kr (S.L.); dpt510@korea.kr (B.-H.L.); 7 Department of Life Sciences, Kyonggi University, Suwon 16227, Korea

**Keywords:** Flaxseed oil, linusorb B3, anti-cancer, apoptosis, actin polymerization, Src, glioblastoma

## Abstract

Linusorbs (LOs) are natural peptides found in flaxseed oil that exert various biological activities. Of LOs, LOB3 ([1–9-NαC]-linusorb B3) was reported to have antioxidative and anti-inflammatory activities; however, its anti-cancer activity has been poorly understood. Therefore, this study investigated the anti-cancer effect of LOB3 and its underlying mechanism in glioblastoma cells. LOB3 induced apoptosis and suppressed the proliferation of C6 cells by inhibiting the expression of anti-apoptotic genes, B cell lymphoma 2 (Bcl-2) and p53, as well as promoting the activation of pro-apoptotic caspases, caspase-3 and -9. LOB3 also retarded the migration of C6 cells, which was achieved by suppressing the formation of the actin cytoskeleton critical for the progression, invasion, and metastasis of cancer. Moreover, LOB3 inhibited the activation of the proto-oncogene, Src, and the downstream effector, signal transducer and activator of transcription 3 (STAT3), in C6 cells. Taken together, these results suggest that LOB3 plays an anti-cancer role by inducing apoptosis and inhibiting the migration of C6 cells through the regulation of apoptosis-related molecules, actin polymerization, and proto-oncogenes.

## 1. Introduction

Glioma is a general term describing brain tumors and includes astrocytic tumors (astrocytomas), oligodendrogliomas, ependymomas, brain stem gliomas, optic pathway gliomas, and mixed gliomas [1]. About one-third of total brain tumors are gliomas originating in the glial cells that surround and support nerve cells in the brain, such as astrocytes, oligodendrocytes, and ependymal cells. Glioblastoma multiforme (GBM), a grade IV astrocytoma, is the most aggressive type of cancer that develops primarily in the brain and spreads into nearby brain tissue. GBM accounts for around 60% of the total primary brain tumors in adults [2]. The annual incidence rate of GBM is up to 5 per 100,000 persons worldwide, and the average survival time is 12–18 months with less than 10% 5-year survival rates after standard treatment [3]. GBM can occur at a broad range of ages but tends to occur more in older adults between the age of 45 and 70, and the mean age for death from brain cancers and other regions of the central nervous system was is 64. GBM diagnosis includes sophisticated imaging techniques, such as computer tomography and magnetic resonance imaging. GBM can be very difficult to treat and a cure is often not possible. Treatments of GBM may slow cancer progression and reduce the signs and symptoms, but there are no known methods to prevent GBM. Current standard treatment usually involves radiation and chemotherapy therapy followed by surgery [4,5,6]. Surgery removes as much of the tumors as possible, but GBM grows into the normal tissue, so complete removal is not possible [7]. Radiation therapy uses high-energy radiation to kill cancer cells and is usually recommended after surgery in the combination with chemotherapy [7]. Chemotherapy uses medications to kill cancer cells. Chemotherapy is also recommended after surgery and is often used during and after radiation therapy [7]. Immunotherapy of GBM is also being studied using programmed cell death protein 1 (PD-1)/PD ligand 1 (PD-L1) immune checkpoint inhibitors [8,9,10]. Preclinical studies in GBM mouse models showed the safety and efficacy, including significant tumor regression and longer survival rate of monoclonal antibody therapeutics targeting PD-1/PD-L1 axis [8]. Currently, monoclonal antibody therapy targeting PD-1/PD-L1 axis is being evaluated in clinical trials concerning GBM patients. However, despite recent medical and surgical advances, treatment of GBM remains very difficult, with poor prognoses and disappointingly low survival rates, and one of the critical concerns of the current chemotherapy is a toxicity issue, which raises the demand for the development of more effective and less toxic medications, such as the natural product-derived complementary and alternative medicines to treat GBM.

Flax (*Linum usitatissimum* L.), also known as linseed, is a fibrous crop and bluish-flowering plant that belongs to the family *Linaceae*. It has been cultivated as a fiber crop and food (flaxseed) in cooler regions of countries, such as Canada, China, and Russia for a long time and is also used in Ayurvedic medicines [11]. Flax is originally cultivated for its fiber, and flax fiber has long been used for manufacturing linen, fabrics, yarn, cordage in many textile industies [12]. Flax is also cultivated for flaxseed. Flaxseed contains 20–25% proteins and 40–45% fatty acids, including the major bioactive ingredients, such as polyunsaturated fatty acids, short-chain omega-3, lignan, mucilage, and linusorbs (LOs) [13]. Flaxseed has been consumed as a dietary supplement for human health and herbal medicines with the purpose of ameliorating many human diseases, including cardiovascular diseases, hypertension, renal disease, cancers, diabetes, stroke, skin disease, gastrointestinal disease, and inflammatory diseases [11,14,15,16,17,18,19,20,21,22,23,24]. Flaxseed has been also used for extracting flaxseed oil that is the oldest commercial oil for foods and pharmaceutical purposes, and flaxseed oil contains many bioactive ingredients such as omega-3 fatty acids, alpha-linolenic acid, lignan secoisolariciresinol diglucoside, and LOs [11,25]. LOs, whose name is derived from *L. usitatissimum*, are natural bioactive orbitide consisting of eight, nine or ten amino acid residues with a molecular weight of approximately 1 kDa and can also be found in flaxseed oil [20,26,27,28]. Studies have demonstrated that LOs have various biological and pharmacological activities, including immunosuppressive, anti-inflammatory, anti-malarial, and anti-cancer effects, [18,20,29,30]. LOB3 ([1–9-NαC]-linusorb B3) was the first LO to be discovered and isolated in flaxseed in 1959 [30] and is the most abundant cyclic nonapeptide. LOB3 and its analogs were reported to have pharmacological properties in disease conditions, such as antioxidative, immunosuppressive, anti-malarial, and anti-inflammatory properties [20,31,32,33]. A few studies have reported that LOB3 also shows cytotoxic activity against several types of cancers [29,34], but its anti-cancer activity, especially anti-GBM activity, and the underlying molecular mechanisms still remain poorly understood. Therefore, the present study investigated the anti-cancer activity of LOB3 and the underlying molecular mechanism in glioblastoma cells.

## 2. Results and Discussion

### 2.1. Cytotoxic and Anti-Proliferative Effect of LOB3 in Cancer Cells

**Figure 1:**
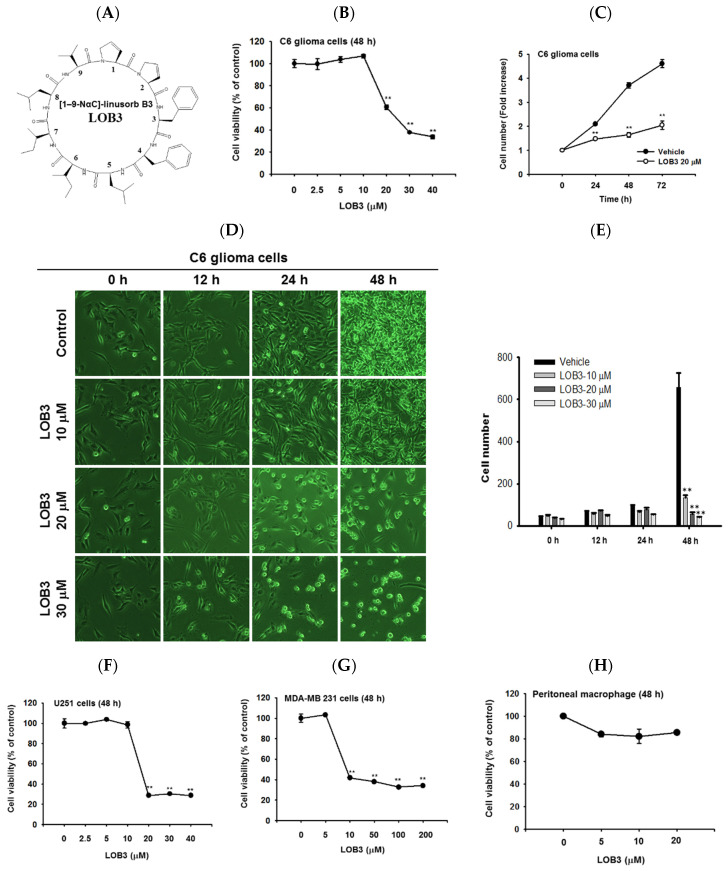
Cytotoxic and anti-proliferative effect of LOB3 in cancer cells. (**A**) Chemical structure of LOB3. (**B**) C6 cells were treated with the indicated doses of LOB3 for 48 h, and cell viability was determined by a conventional 3-(4,5-dimethylthiazol-2-yl)-2,5-diphenyltetrazolium bromide (MTT) assay. (**C**) C6 cells were treated with LOB3 (20 μM) for the indicated time, and viable cell numbers were determined by a conventional MTT assay. (**D**,**E**) C6 cells were treated with the indicated doses of LOB3 for the indicated time, and cell numbers and shapes were observed under a light microscope. Photos of the cells were taken by a digital camera (**D**) and numbers of cells were counted by a cell counter (**E**). (**F**–**H**) U251, MDA-MD-231, and peritoneal macrophage cells were treated with the indicated doses of LOB3 for 48 h, and cell viability was determined by a conventional MTT assay. The data (**B**,**C**,**E**,**F**–**H**) are expressed as the means ± standard error of the mean (SEM) of three independent experiments. Statistical significance was analyzed by the Mann-Whitney U test. ** *p* < 0.01 compared to the vehicle-treated controls.

LOs are small biologically active cyclic peptides found in flaxseed oil, and many types of LOs have been identified and named based on their structures [35]. Of LOs, LOB3 (Figure 1A, molecular weight: 1040.34) and its analogs have been demonstrated to play pivotal roles in antioxidative [31] and anti-inflammatory actions [20]. Interestingly, recent studies have reported the cytotoxic effect of LOB3 on cancer cells [29,34,36]; however, the anti-cancer effect of LOB3 and the underlying mechanisms are poorly understood. Therefore, this study investigated the anti-cancer activity of LOB3 and the underlying mechanisms in C6 glioblastoma cells, since glioblastoma multiforme is the most aggressive type of brain cancer with a high recurrence rate and a low 5-year survival rate [37].

First, the cytotoxic effect of LOB3 was evaluated in C6 cells. C6 cells were treated with various doses of LOB3 for 48 h, and cell viability was examined by an MTT [3-(4,5-dimethylthiazol-2-yl)-2,5-diphenyltetrazolium bromide] assay. LOB3 from 20 to 40 μM exerted a cytotoxic effect in C6 cells in a dose-dependent manner, while no cytotoxic effect of LOB3 was shown at doses lower than 10 μM (Figure 1B). One of the fundamental features of cancer is tumor clonality and uncontrolled proliferation. Therefore, the anti-proliferative effect of LOB3 was also evaluated in C6 cells. C6 cells treated with LOB3 (20 μM) were cultured for 72 h, and the proliferation rate of LOB3-treated C6 cells was significantly reduced compared to that of the vehicle-treated control cells (Figure 1C). These results were confirmed by observing the shape and the numbers of C6 cells after LOB3 treatment. Similar to the results depicted in Figure 1B,C, LOB3 exerted a cytotoxic effect in C6 cells by changing the cell shape and reducing cell numbers at 20 and 30 μM in a time- and dose-dependent manner (Figure 1D,E).

The cytotoxic effect of LOB3 on cancer cells was further investigated in another glioblastoma cell line, U251 cells, and a breast cancer cell line, MDA-MB-231 cells. Similar to the C6 cells, LOB3 significantly reduced the viability of U251 cells at doses of 20 μM and greater, but no cytotoxic effect was observed at doses lower than 10 μM (Figure 1F). Similar to a previous study [34], LOB3 also induced the cytotoxicity of MDA-MB-231 cells, but MDA-MB-231 cells were more sensitive to LOB3. LOB3 exerted the cytotoxic effect in the breast cancer cells at doses as low as 10 μM (Figure 1G), while the two glioblastoma cell lines were sensitive and dead at 20 μM (Figure 1B,C,F), indicating that the drug sensitivity and cytotoxic effect of LOB3 on cancer cells depend on the types of cancer cells. Furthermore, cytotoxicity of this compound was not found in non-malignant cells, peritoneal macrophages (Figure 1H). Similarly, other natural bioactive orbitides such as surfactins and beauvericin displayed anti-cancer activity in giant-cell tumors of the bone (GCTB) cells, MCF-7 breast tumor cells, and CT-26 lymphoma [38,39,40], implying that cytotoxic activity of LOB3 might be due to structural feature of this compound. Taken together, these results suggest that LOB3 plays an anti-cancer role by inducing cytotoxicity and reducing the proliferation of cancer cells).

### 2.2. Cytotoxic Effect of LOB3 on C6 Cells by Apoptosis

**Figure 2:**
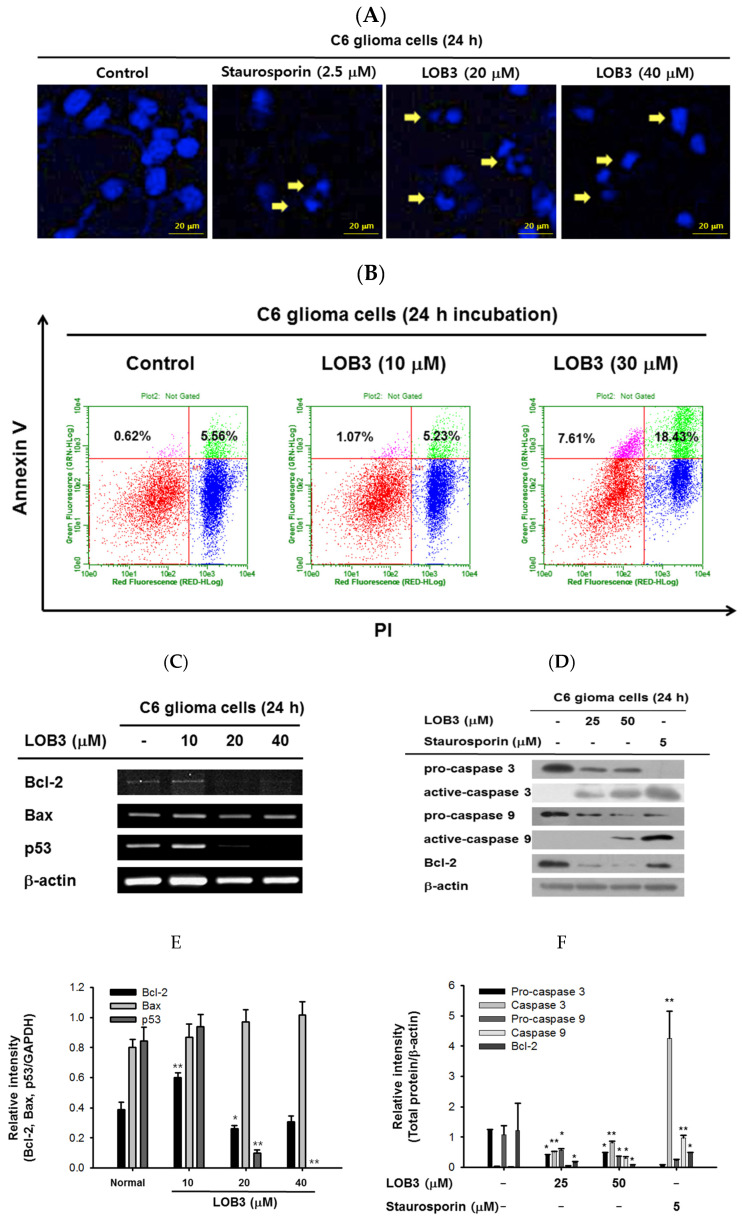
Cytotoxic effect of LOB3 on C6 cells by apoptosis. (**A**) The nuclei of C6 cells treated with either staurosporine (2.5 μM) or LOB3 (20 and 30 μM) were stained with Hoechst 33342 and observed under a fluorescence microscope. Yellow arrows indicate nuclear shrinking and fragmentation. (**B**) C6 cells treated with the indicated doses of LOB3 for 24 h were stained with PI and annexin V-FITC, and the cell population was determined by flow cytometry analysis. (**C**) C6 cells were treated with the indicated doses of LOB3 for 24 h, and mRNA levels of Bcl-2, BAX, and p53 were analyzed by semiquantitative RT-PCR. (**D**) C6 cells were treated with either staurosporine (5 μM) or LOB3 (25 and 50 μM) for 24 h, and protein levels of pro-caspase-3, caspase-3, pro-caspase-9, and caspase-9 were determined by Western blot analysis. The data (**E**,**F**) are expressed as the means ± standard deviation (SD) of three experiments. Statistical significance was analyzed by the Mann-Whitney U test. Results (**A**,**B**). Data of band intensity (**E**,**F**) were measured and quantified using ImageJ. * *p* < 0.05 and ** *p* < 0.01 compared to the vehicle-treated controls.

Apoptosis is a form of programmed cell death occurring in multicellular organisms and is characterized by many biochemical events leading to cell changes and eventually death [41,42]. surfactins and beauvericin were reported to induce apoptosis in cancer cells [36,38,43]; therefore, whether the cytotoxic effect of LOB3 on cancer cells is mediated by apoptosis was evaluated in C6 cells. One of the major characteristics of apoptosis is nuclear shrinking and fragmentation [44,45], and these events were examined in LOB3-treated C6 cells by Hoechst nuclear staining. Compared to the control, LOB3 (20 and 30 μM) had a similar effect on C6 cells as staurosporine, an apoptosis inducer [46], in that it stimulated nuclear shrinking and fragmentation (Figure 2A). Double staining of annexin V and propidium iodide (PI) is a commonly used analytical approach for detecting apoptosis of cells [47], and this method was used to examine LOB3-induced apoptosis of C6 cells. The proportions of early and late apoptosis of LOB3-treated C6 cells were quantified by flow cytometry analysis after Annexin V/PI staining, and the results indicated that LOB3 significantly induced the apoptotic population of C6 cells at doses of 30 μM, but not 10 μM (Figure 2B). This result is consistent with the result that LOB3 exhibited the cytotoxicity-inducing effect from doses of 20 μM (Figure 1B,D).

The mechanism by which LOB3 exhibited an apoptotic effect on C6 cells was next evaluated at a molecular level. Bcl-2 family members play pivotal roles in the regulation of apoptosis and are categorized into two major groups: anti-apoptotic members, including Bcl-2, Bcl-X_L_, Bcl-W, MCL-1, and BFL-1/A1, and pro-apoptotic members, including BAX, BAK, BOK, and BAD [48]. The effect of LOB3 on the mRNA expression of both anti-apoptotic and pro-apoptotic Bcl-2 family members was examined in C6 cells. LOB3 decreased mRNA expression of the anti-apoptotic member Bcl-2 at doses of 20 and 40 μM (Figure 2C–F) but showed no marked effect on mRNA expression of the pro-apoptotic member BAX at all doses in C6 cells (Figure 2C,E), suggesting that LOB3 induced apoptotic death of C6 cells by inhibiting the expression of the anti-apoptotic member, Bcl-2 rather than increasing the expression of the pro-apoptotic member, BAX. p53 is a tumor suppressor, but strong evidence has accumulated to indicate that p53 plays an anti-apoptotic role by transcriptionally activating many genes whose products efficiently suppress apoptosis [49]. Therefore, the effect of LOB3 on mRNA expression of p53 was examined, and mRNA expression of p53 was markedly decreased in the LOB3-treated C6 cells at doses of 20 and 40 μM (Figure 2C,E).

Caspases are a family of endonucleases that act as critical links in the molecular networks that control apoptosis and play critical roles in the induction of apoptosis as both initiators (caspase-2, -8, -9, -10, 20, and -22) and executioners (caspase-3, -6, -7, and 21) [50,51]. Caspases are initially expressed as inactive procaspases that are activated by pro-apoptotic signals via proteolytic cleavage. Therefore, the effect of LOB3 on the proteolytic activation of caspases was examined in C6 cells. LOB3 activated both an apoptosis initiator, caspase-9, as well as an executioner, caspase-3, by promoting the proteolytic cleavage of these caspases at doses of 25 and 50 μM in C6 cells (Figure 2D,F), indicating that LOB3 induces apoptosis of C6 cells by activating the apoptosis initiators and executioners. Moreover, similar to the semi-quantitative RT-PCR result (Figure 2C,E), LOB3 markedly reduced the protein expression of the anti-apoptotic Bc1 family member, Bcl-2, at doses of 25 and 50 μM in C6 cells (Figure 2D,F). Taken together, these results suggest that the cytotoxic effect of LOB3 on C6 cells is mediated by the induction of apoptosis through inhibiting the expression of anti-apoptotic genes, such as Bcl-2 and p53, as well as activating the proteolytic processing of both apoptosis initiator, caspase-9, and executioner, caspase-3.

### 2.3. Anti-Migratory Effect of LOB3 in C6 Cells

**Figure 3:**
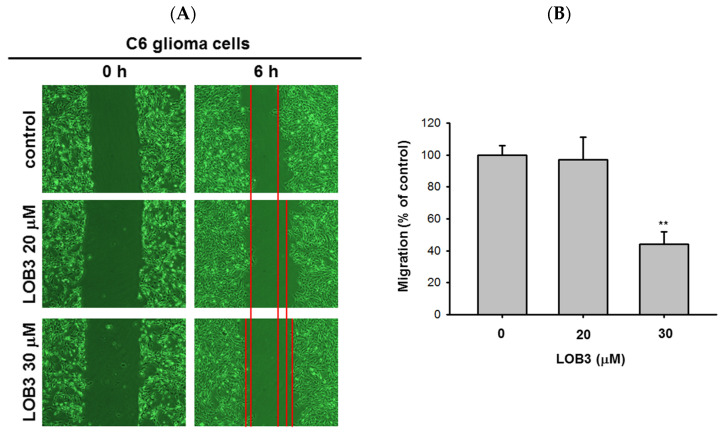
Anti-migratory effect of LOB3 in C6 cells. (**A**) Migration of C6 cells treated with the indicated doses of LOB3 for 6 h was analyzed under a light microscope. (**B**) Migration areas (% of control) were calculated and plotted with Figure 3A. Statistical significance was analyzed by the Mann-Whitney U test. Result (**A**) is a representative of three experiments. The data (**B**) are expressed as the means ± SD of three experiments. ** *p* < 0.01 compared to the vehicle-treated controls.

Another fundamental feature of cancer is the migration of tumor cells from the original location where tumors arise to other parts of the body. It was reported that surfactin can reduce the 12-*O*-tetradecanoylphorbol-13-acetate (TPA)-induced metastatic potentials, including invasion and migration of human breast carcinoma cells [52]. Therefore, the anti-migratory effect of LOB3 was evaluated in C6 cells. C6 cells treated with LOB3 (20 and 30 μM) were cultured for 6 h, and the degree of C6 cell migration was examined. Compared to the control, LOB3 markedly suppressed the migration of C6 cells at a dose of 30 μM (Figure 3A,B). Interestingly, although LOB3 exerted a cytotoxic and anti-proliferative effect on C6 cells (Figure 1B–D) by facilitating apoptosis at doses as low as 20 μM (Figure 2), LOB3 did not suppress the migration of C6 cells at 20 μM, but 30 μM. This result indicates that the doses of LOB3 required to inhibit the proliferation and migration of C6 cells might not be same, since the molecules critical for cell proliferation and migration are different, and the inhibitory effect of LOB3 on the biological actions of these molecules might also be different in C6 cells. To identify the effective and optimal doses targeting both sets of these molecules and thereby inhibiting both the proliferation and migration of C6 cells, further molecular mechanism studies using various doses of LOB3 will be required. Taken together, these results suggest that LOB3 plays an anti-cancer role by not only inducing cytotoxicity but also suppressing the migration of C6 cells.

### 2.4. Inhibitory Effect of LOB3 on Actin Polymerization in C6 Cells through the Targeting of Src and STAT3

**Figure 4:**
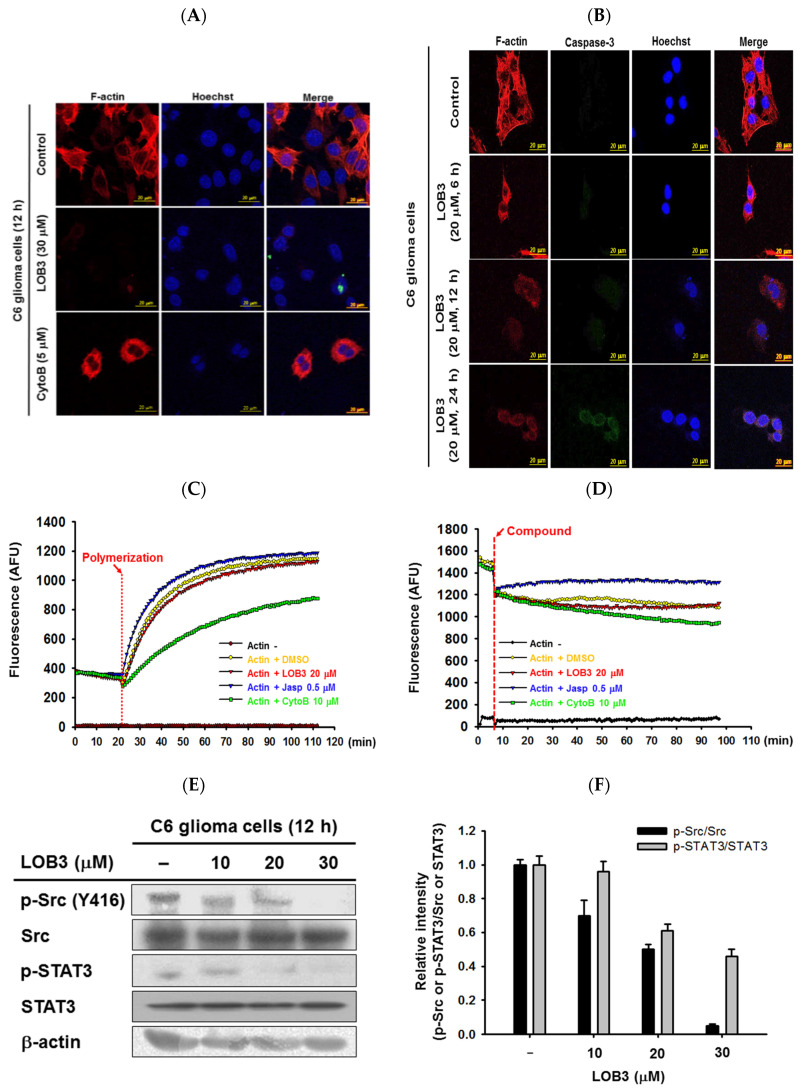
Inhibitory effect of LOB3 on actin polymerization by targeting Src and STAT3 in C6 cells. (**A**) Actin filaments (F-actin) and the nuclei of C6 cells treated with either LOB3 (30 μM) or CytoB (5 μM) for 12 h were stained with phalloidin and Hoechst 33342, respectively, and visualized under a confocal microscope. (**B**) Actin filaments (F-actin), caspase-3, and the nuclei of C6 cells treated with LOB3 (20 μM) for the indicated time were stained with phalloidin, caspase-3 antibody, and Hoechst 33342, respectively, and visualized under a confocal microscope. (**C**) Actin monomers were incubated with the indicated compounds for the indicated time, and actin polymerization was analyzed by an in vitro actin polymerization assay. (**D**) Actin filaments were incubated with the indicated compounds for the indicated time, and actin depolymerization was analyzed by an in vitro actin de-polymerization assay. (**E**) C6 cells were treated with the indicated doses of LOB3 for 12 h, and the protein levels of the phosphor and total forms of Src and STAT3 were determined by Western blot analysis. Results (**A**–**D**) are representative of three independent experiments. Statistical significance was analyzed by the Mann-Whitney U test. Data of band intensity (**F**) were measured and quantified using ImageJ. * *p* < 0.05 and ** *p* < 0.01 compared to the vehicle-treated controls.

The cytoskeleton is a dynamic and complex intracellular network of protein filaments interlinking in the cytoplasm of cells, and its primary function is to provide the cells with their shape and with mechanical resistance to deformation stresses. Of the three main components of the cytoskeleton, the actin cytoskeleton is essential to enabling cell motility by maintaining the shape and integrity of the cell. In addition, the actin cytoskeleton plays a critical role in the migration, invasion, and metastasis of cancer cells, as well as overall cancer progression [53,54,55,56]; therefore, the selective and effective targeting of actin in cancer cells is a worthwhile strategy in the development of anti-cancer therapeutics [56,57,58,59,60]. The effect of LOB3 on the actin cytoskeleton was examined in C6 cells. C6 cells treated with LOB3 or cytochalasin B (CytoB), an actin polymerization inhibitor [61], were incubated with phalloidin to visualize filamentous actin (F-actin) [62]. Although CytoB moderately inhibited the formation of the actin cytoskeleton, LOB3 (30 μM) dramatically inhibited the formation of the actin cytoskeleton in C6 cells (Figure 4A). Since LOB3 almost completely inhibited the formation of the actin cytoskeleton in C6 cells at 30 μM (Figure 4A), the inhibitory effect of LOB3 on the formation of the actin cytoskeleton in C6 cells was further examined at a lower dose (20 μM) for different time periods. LOB3 (20 μM) also markedly inhibited the formation of the actin cytoskeleton at 12 h and 24 h after treatment, while LOB3 (20 μM) moderately inhibited the formation of the actin cytoskeleton at 6 h after treatment (Figure 4B). This result does not necessarily mean LOB3 needs at least 6 h to inhibit the formation of the actin cytoskeleton in C6 cells, and the time required to inhibit actin cytoskeleton formation might vary depending on LOB3 doses. As discussed earlier, caspase-3 is an executioner of apoptosis [35,36], and similar to the previous result (Figure 2D), LOB3 (20 μM) induced proteolysis of pro-caspase-3 and produced active caspase-3 at 24 h in C6 cells (Figure 4B).

The effect of LOB3 on the formation of the actin cytoskeleton was further evaluated by in vitro actin polymerization and depolymerization assays. LOB3 (20 μM) suppressed actin polymerization, while jasplakinolide (Jasp, 0.5 μM), an actin polymerization promoting peptide [63], and CytoB (10 μM) induced and suppressed actin polymerization, respectively (Figure 4C). Similarly, LOB3 (20 μM) promoted actin de-polymerization, while Jasp (0.5 μM) and CytoB (10 μM) inhibited and promoted actin depolymerization, respectively (Figure 4D). LOB3 suppressed the migratory ability of C6 cells (Figure 3), and this suppressive effect of LOB3 on C6 cell migration could be achieved by the suppression of actin polymerization. Taken together, these results suggested that LOB3 played an anti-cancer role by suppressing actin polymerization as well as promoting actin depolymerization.

Src is a proto-oncogene that is strongly implicated in the growth, progression, invasion, and metastasis of many types of human cancers [64,65]. Interestingly, actin polymerization induces Src activation with delivery to the cell membrane [66]. Since LOB3 played an inhibitory role in actin polymerization (Figure 4A–D), whether LOB3 suppresses Src activation was next examined in C6 cells. LOB3 suppressed Src activation in C6 cells in a dose-dependent manner (Figure 4E,F), thereby indicating that LOB3 suppresses the activation of a proto-oncogene, Src, by inhibiting actin polymerization in C6 cells. Src regulates various downstream signaling pathways in cancer cells, leading to the development and progression of cancers, and one of the most critical downstream target molecules is STAT3 [67,68,69]. Therefore, whether LOB3 also suppresses the activation of STAT3 was examined, and as expected, LOB3 was found to suppress STAT3 activation in C6 cells (Figure 4E,F). Taken together, LOB3-induced anti-cancer activity in C6 cells is mediated by the inhibition of actin polymerization and the subsequent suppression of Src and the downstream molecule, STAT3.

## 3. Materials and Methods

### 3.1. Materials

LOB3 (Figure 1A) was provided as a generous contribution from Prairie Tide Diversified Inc. (Saskatoon, SK, Canada). The C6 human glioblastoma cell line, U251 human glioblastoma cell line, and MDA-MB-231 human breast cancer cell line were purchased from the American Type Culture Collection (Rockville, MD, USA). Dulbecco’s modified Eagle’s medium (DMEM), fetal bovine serum (FBS), phosphate-buffered saline (PBS), penicillin, streptomycin, L-glutamine, bovine serum albumin (BSA), apoptosis analysis kit (Dead Cell Apoptosis Kit with Annexin V FITC and PI), MuLV reverse transcriptase (RT), and Lipofectamine^®^ 2000 reagent were purchased from Thermo Fisher Scientific (Waltham, MA, USA). MTT, Hoechst 33342, staurosporine, cytochalasin B (CytoB), and jasplakinolide (Jasp) were purchased from Sigma-Aldrich (St. Louis, MO, USA). TRI reagent^®^ was purchased from Molecular Research Center, Inc. (Cincinnati, OH, USA). Primers for semi-quantitative RT polymerase chain reaction (PCR) were synthesized, and PCR premix was purchased from Bioneer, Inc. (Daejeon, Korea). An enhanced chemiluminescence system was purchased from AbFrontier (Seoul, Korea). Antibodies specific to each target used for Western blot analysis and immunofluorescence staining were purchased from Cell Signaling Technology (Beverly, MA, USA) and Santa Cruz Biotechnology (Santa Cruz, CA, USA). The Actin Polymerization Biochem kit was purchased from Cytoskeleton (Denver, CO, USA).

### 3.2. Preparation of Peritoneal Macrophages

Peritoneal exudates were isolated from ICR mice (6-week-old, 17 to 21 g) by lavage 4 days after intraperitoneal treatment with 4% thioglycolate broth (Difco Laboratories, Detroit, MI, USA). After the blood was removed from the exudates using RBC lysis buffer (Sigma Chemical Co., St. Louis, MO, USA), the extracted peritoneal macrophages (1 × 10^6^ cells/mL) were plated in a 100 mm tissue culture plate and incubated for 4 h at 37 °C in a 5% CO_2_ humidified atmosphere. The ICR male mice were purchased from Daehan Bio Link Co., Ltd. (Chungbuk, Korea) and housed at seven mice per group under a 12-h light/dark cycle (lights on at 6 a.m.). Water and a pellet diet (Samyang, Daejeon, Korea) were supplied ad libitum. Animal care followed guidelines issued by the National Institutes of Health for the Care and Use of Laboratory Animals (NIH Publication 80–23, revised in 1996) and the Institutional Animal Care and Use Committee at Sungkyunkwan University (Suwon, Korea).

### 3.3. Cell Culture

C6 cells, U251 cells, and MDA-MB-231 cells as well as peritoneal macrophages were cultured or maintained in DMEM supplemented with 10% FBS, penicillin (100 U/mL), streptomycin (100 mg/mL), and L-glutamine (2 mM) at 37 °C in a humidified incubator with 5% CO_2_. Cells were kept fresh by splitting them 2–3 times per week.

### 3.4. Cell Proliferation and Viability Assay

C6, U251, and MDA-MB-231 cells as well as peritoneal macrophages were treated with LOB3 at the indicated doses and time periods, and cell viability was determined by a conventional MTT assay [70,71]. For an MTT assay, the MTT solution was incubated with the cells at 37 °C for 4 h, and then stop solution (10% SDS in 0.01 N HCl) was added to the cells. After incubation at 37 °C for 24 h, the optical density was determined at 540 nm using a microplate reader (BioTek, Winooski, VT, USA).

### 3.5. Cell Death Assays and Flow Cytometry Analysis

The death of C6 cells was also evaluated by observing their shapes. C6 cells were treated with LOB3 (0, 10, 20, and 30 μM) for 0, 12, 24, and 48 h, and the shapes of the cells were observed and evaluated under a light microscope. The death of C6 cells was also analyzed by Hoechst 33342 (10 μg/mL in PBS) staining. C6 cells were stained with Hoechst 33342 at room temperature for 30 min and washed with PBS three times. Hoechst 33342-stained nuclei were observed and analyzed under a fluorescence microscope. The apoptotic death of C6 cells was evaluated by flow cytometry analysis using an apoptosis analysis kit (see Materials) according to the manufacturer’s instructions. Briefly, C6 cells pretreated with LOB3 (0, 10, and 30 μM) for 24 h were incubated with propidium iodide (PI) and annexin V-FITC in a binding buffer (50 mM HEPES, 700 mM NaCl, 12.5 mM CaCl_2_, pH 7.4) at room temperature for 15 min. After washing the cells with cold PBS three times, the population of fluorescent cells was determined by flow cytometry analysis.

### 3.6. Semi-Quantitative RT-PCR

Total RNA was extracted from the C6 cells treated with LOB3 (0, 10, 20, and 40 μM) for 24 h using TRI reagent^®^, followed immediately by the synthesis of cDNA from total RNA (1 μg) using MuLV RT according to the manufacturer’s instructions. Semi-quantitative RT-PCR was conducted using the cDNA to determine the mRNA expression levels of Bcl-2, Bax, and p53. The experimental conditions and the primer sequences used for the semi-quantitative RT-PCR in this study are listed in Table 1 and Table 2, respectively.

### 3.7. Western Blot Analysis

Whole lysates of C6 cells treated with the indicated concentration of LOB3 or staurosporine (5 μM) for the indicated time were prepared by lysing the cells using radioimmunoprecipitation assay (RIPA) buffer (50 mM Tris-HCl pH 8.0, 150 mM NaCl, 1% Nonidet P-40, 0.5% sodium deoxycholate, and 0.1% sodium dodecyl sulfate [SDS]) containing proteinase inhibitors (1 mM sodium orthovanadate, 10 μg/mL aprotinin, 10 μg/mL pepstatin, 1 mM benzamide, and 2 mM PMSF) in ice for 30 min, followed by sonication for 10 sec. Whole lysates of HEK293 cells treated with LOB3 (0, 20, and 40 μM) and transfected with either empty plasmids (pcDNA) or HA-Src plasmids for 12 h were also prepared according to the same method as was used with the C6 cells. Sample buffer (62.5 mM Tris-HCl pH 6.8, 2.5% SDS, 0.002% bromophenol blue, 5% β-mercaptoethanol, 10% glycerol) was added to the whole cell lysates, followed by boiling for 10 min. For Western blot analysis, the whole cell lysates were subjected to SDS-polyacrylamide electrophoresis and transferred to polyvinylidene difluoride membrane (250 mA for 1 h) in transfer buffer (25 mM Tris, 192 mM glycine, pH 8.3, 20% methanol (*v*/*v*)). Targets (pro-caspase-3 and -9, caspase-3 and -9, Bcl-2, p-Src [Y416], p-Src [Y527], Src, p-STAT3, STAT-3, and HA) were detected with their specific primary (1:1000) and secondary (1:15,000) antibodies, and the immune complexes were visualized using an enhanced chemiluminescence system according to the manufacturer’s instructions as reported previously [71].

### 3.8. In Vitro Cell Migration Assay

C6 cells grown to a confluent monolayer were treated with LOB3 (0, 20, and 30 μM) and scratched using a pipette tip as previously described with slight modification [72,73]. After 6 h, the migrated cells to the scratched regions were observed and taken pictures under a light microscope (Olympus, Japan). The migrated C6 cells were measured using ImageJ software (NIH, Bethesda, MD, USA), and compared by plotting (% control).

### 3.9. Confocal Microscopy

C6 cells were treated with either LOB3 (0, 20, and 30 μM) or CytoB (5 μM) for the indicated time. For confocal microscopic analysis, the cells were fixed with 4% paraformaldehyde in PBS for 10 min and permeabilized with 0.5% Triton X-100 in PBS for 10 min. The cells were next blocked with 1% BSA in PBS for 1 h, followed by incubation with Rhodamine Phalloidin reagent or the antibodies specific for F-actin and cleaved caspase-3 at 4°C overnight. The cells were then incubated with Alexa Fluor 488-or 568-conjugated secondary antibodies for 1 h. The DNA of these cells was stained with Hoechst 33342 (10 μg/mL in PBS) for 30 min, followed by washing with PBS for 5 min three times. The cells were mounted on the glass slides and imaged using a laser-scanning confocal microscope (Zeiss LSM 710 META, Oberkochen, Germany) with a 63× oil-immersion objective lens.

### 3.10. In Vitro Actin Polymerization and Depolymerization Assays

Actin polymerization and depolymerization assays were conducted in the presence and absence of either LOB3 (20 μM), Jasp (0.5 μM), or CytoB (10 μM), using the Actin Polymerization Biochem kit according to the manufacturer’s instructions. For actin polymerization assay, LOB3, Jasp, or CytoB were mixed with pyrene-labeled globular actin (G-actin) in actin polymerization buffer, and the fluorescence was measured for 90 min using a fluorescence microplate reader (BioTek, Winooski, VT, USA). For actin depolymerization assay, LOB3, Jasp, or CytoB were incubated with pyrene-labeled F-actin in depolymerization buffer, and the fluorescence was measured for 90 min using a fluorescence microplate reader. The effect of each compound on actin polymerization and de-polymerization was determined by fluorescence of pyrene-labeled actin, measured with an excitation wavelength of 350 nm and an emission wavelength of 407 nm at 25 °C every 60 s for 1 h.

### 3.11. Statistical Analysis 

Data (Figure 1B,C,E–G) are presented as the mean ± standard error of the mean (SEM) of three independent sets of experimental data performed with at least three samples. The data (Figure 1D right panel, bottom panels of Figure 2C,D, Figure 3B, and Figure 4E right panel) are expressed as the means ± standard deviation (SD) of three experiments. Statistical differences between groups in these data were analyzed by the Mann-Whitney U test, and *p*-value < 0.05 was considered to indicate a statistically significant difference. All statistical analyses were conducted using SPSS software (SPSS Inc., Chicago, IL, USA). Other results are representative of at least two of the data sets.

## 4. Conclusions

**Figure 5:**
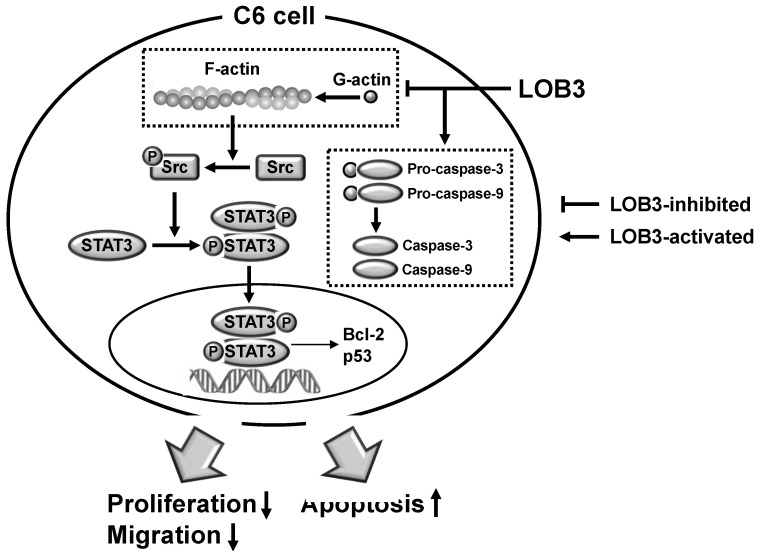
The proposed model to illustrate the anti-cancer activity of LOB3 in a C6 cell.

The current study investigated the anti-cancer effect of LOB3 and the underlying molecular mechanism in glioblastoma C6 cells. LOB3 induces the cytotoxicity of C6 cells by promoting apoptosis through modulating the expression of apoptosis-related genes and molecules. LOB3 also suppressed the motility of C6 cells, which is critical for cancer cell migration, invasion, and metastasis, by inhibiting actin polymerization, and LOB3 suppressed the activation of Src and STAT3, which are proto-oncogenic factors activated by actin polymerization in cancer cells. Despite these results, this study was limited to in vitro experiments using cancer cell lines, and further ex vivo studies using tumor cells from cancer animal models or human patients as well as in vivo studies using animal xenograft or orthotopic models are required to support and confirm the results of this study. In addition, the anti-cancer effect of LOB3 needs to be expanded to other types of cancers to confirm the general anti-cancer effect of LOB3 in a broad range of cancers. Moreover, the comparison studies for the anti-cancer effect of various LOs also need to be further investigated. In conclusion, LOB3 plays an anti-cancer role by facilitating the apoptotic death of C6 cells as well as inhibiting the migratory activity of C6 cells by modulating multiple factors associated with apoptosis, motility, cytoskeleton formation, and proto-oncogenic functions, as described in Figure 5. Given this evidence, this study proposes an anti-cancer role of a cyclic peptide, LOB3, which is present in flaxseed oil, in glioblastoma cells with a new understanding of the underlying molecular mechanisms, which could provide insight into the development of effective and safer LO-based therapeutics to prevent and treat glioblastoma and even other types of cancers.

## Figures and Tables

**Table 1:** The experimental conditions of the semi-quantitative RT-PCR in this study.

Targets	Annealing Temp.	Cycle No.	Fragment Size (Base Pair)
Bcl-2	60	30	304
Bax	60	30	240
p53	60	30	560
GAPDH	60	25	350

**Table 2:** Primer sequences used for the semi-quantitative RT-PCR in this study.

Targets		Sequences (5′ to 3′)
Bcl-2	Forward	CACCCCTGGCATCTTCTCCTT
	Reverse	CACAATCCTCCCCCAGTTCACC
Bax	Forward	ATGGCTGGGGAGACACCTGAG
	Reverse	CTAGCAAAGTAGAAAAGGGCAAC
p53	Forward	CTCTGTCATCTTCCGTCCCTTC
	Reverse	AGGACAGGCACAAACACGAAC
GAPDH	Forward	CACTCACGGCAAATTCAACGGCAC
	Reverse	GACTCCACGACATACTCAGCAC
